# Reply to Letter: Real‐World Clinical Experience With Serum MOG and AQP4 Antibody Testing by Live Versus Fixed Cell‐Based Assay

**DOI:** 10.1002/acn3.70061

**Published:** 2025-05-14

**Authors:** Yana Said, Conlan Tran, LuAnn Rezavi, Patrizio Caturegli, Eoin P. Flanagan, Elias S. Sotirchos

**Affiliations:** ^1^ Department of Neurology Johns Hopkins University School of Medicine Baltimore Maryland USA; ^2^ Department of Pathology Johns Hopkins University School of Medicine Baltimore Maryland USA; ^3^ Departments of Neurology, Laboratory Medicine and Pathology, and Center for MS and Autoimmune Neurology Mayo Clinic College of Medicine Rochester Minnesota USA

**Keywords:** antibody testing, APQ4, MOG, MOGAD, NMOSD

We thank Dr. Budhram for their interest in our manuscript and for raising these important questions [1].

First, as discussed in our paper, we would like to note that our results comparing fixed CBA (FCBA) vs. live CBA (LCBA) for MOG‐IgG testing are remarkably similar to a large study by Tea et al., who found that only 56% (78/139) of pediatric and 53% (79/148) of adult MOG‐IgG LCBA‐positive sera were able to bind to fixed MOG [2, 3]. Furthermore, for AQP4‐IgG testing, a prior comparative multi‐center study found differences in sensitivity for LCBA vs. FCBA ranging from ~5% to 15% (lower for FCBA) [4]. While we found a larger discrepancy in sensitivity, it should be noted that in that study sera from NMO/NMOSD patients were provided for comparative testing by several centers, including by centers that utilized FCBA for testing AQP4‐IgG. While centers provided both AQP4‐IgG seropositive and seronegative samples (based on their in‐house testing), a limited number of samples were selected by each center (rather than providing all available NMO/NMOSD sera), raising the possibility of potential selection bias to include a higher proportion of AQP4‐IgG seropositive samples by FCBA by centers utilizing these assays, which would be expected to inflate FCBA sensitivity.

At the Johns Hopkins Immunology Laboratory, the fixed CBAs are performed by experienced technologists using EuroImmun's MOG transfected cell slides (FA 1156‐1010‐50) and EuroImmun's AQP4‐transfected cell slides (FA 1128‐1010‐50). The slides are prepared according to the manufacturer's recommendations and screened at 1:10 dilution. After staining, the slides are read manually using a Nikon Eclipse 80i fluorescence microscope, fitted with an FITC (Nikon 96302) filter. Samples determined to be potentially positive at the screening dilution are repeated at 1:10 and serially diluted up to 1:640. Positive wells are then graded 1+ to 4+, and the last titer demonstrating a 1+ fluorescence is reported as the end titer. Screening slides are read by a single reader, and any potential positives that are repeated and diluted are then read by the original reporter and reviewed by a second reader. The last titer demonstrating 1+ positivity is agreed upon by both readers before reporting. The Johns Hopkins Immunology Laboratory also performs the Euroimmun Autoimmune Encephalitis Mosaic (FC 112d‐1005‐6) which includes cells transfected to express NMDA, CASPR2, AMPA1/2, LGI1, DPPX, and GABA‐B receptor. Moreover, this laboratory has extensive experience with other indirect immunofluorescence tests including ANA using HEp‐2 cells, dsDNA antibodies by Crithidia Luciliae, and other antibody testing using whole tissues, such as mouse kidney, liver, and stomach tissues, and monkey esophagus. Furthermore, driven by the results of this study, the Johns Hopkins Immunology Laboratory developed a MOG‐IgG1 LCBA using transiently transfected HEK293 cells and analyzed by flow cytometry and has phased out use of the Euroimmun MOG‐IgG FCBA. Notably, when performing validation of the sensitivity of this new in‐house LCBA, similar discrepancies were found in the performance Euroimmun FCBA compared to the LCBA as those that we noted previously in our study [2].

With respect to the question concerning indications for testing by both assays, it is not possible to determine in each case what the exact rationale was, as testing was ordered during the examined period (July 2019–January 2024) by > 50 different ordering physicians, with experience levels ranging from residents to attendings, and physician specialties including general neurology, neuroimmunology, ophthalmology, neuro‐ophthalmology, and rheumatology. In general, samples from cases with high enough clinical suspicion (per the ordering physician) had both tests ordered simultaneously, or if negative by the in‐house FCBA, were then sent for confirmation with the LCBA using leftover serum. While positive results from FCBA were retested by LCBA in many cases for confirmation of the positive result, this appeared to be somewhat less frequently done if the positive result was consistent with a clinical phenotype of MOGAD or NMOSD. As Dr. Budhram astutely points out, this led to an enrichment in patients who tested positive only by a single assay (usually the FCBA) in the samples derived from the biorepository, which was expected given the above selection bias.

In order to address the concerns raised by Dr. Budhram regarding the potential influence of this selection bias on the estimates of sensitivity for each assay, we have performed additional analyses including all samples that tested positive by the FCBA, under the hypothetical scenario that they were also tested on the same day by the LCBA, in order to obtain unbiased estimates of the sensitivity of each assay over the examined time period. Overall, there were 2076 MOG‐IgG FCBA tests performed at Johns Hopkins Hospital between July 2019 and January 2024, of which 594 were tested by LCBA on the same day and included in our original study [2]. Of the remaining 1482 samples, 34 samples from 34 unique patients tested positive for MOG‐IgG by FCBA, with 27/34 fulfilling diagnostic criteria for MOGAD. The revised estimated sensitivity for the MOG‐IgG FCBA with these samples also included in the analysis would be 59.3% vs. the previously reported estimate of 45.7%. Assuming that only ~90% of these 27 samples (24 samples) would have tested positive by MOG‐IgG LCBA on the same day (a rather conservative estimate based on what we and others have found in comparative analyses of sensitivity of MOG FCBA vs. LCBA), the estimated sensitivity of MOG‐IgG LCBA would be 93.5% vs. the previously reported estimate of 95.1%. Notably, while these sensitivity estimates are improved for MOG‐IgG FCBA, they remain markedly discrepant between the two assays. Furthermore, this is a “best‐case” scenario for the MOG‐IgG FCBA, as it ignores the possibility of the remaining samples that were “negative” (tested exclusively by MOG‐IgG FCBA) being positive by the LCBA in patients fulfilling MOGAD criteria (a scenario that we found to be quite common when samples were tested on the same day, as evidenced by the markedly superior sensitivity of LCBA).

For AQP4‐IgG testing, there were 1729 AQP4‐IgG FCBA tests performed at Johns Hopkins Hospital between July 2019 and January 2024, of which 577 were tested by LCBA on the same day and included in our original study [2]. Of the remaining 1152 samples, 18 samples from 18 unique patients tested positive for AQP4‐IgG by FCBA, with all fulfilling 2015 IPND diagnostic criteria for AQP4‐IgG seropositive NMOSD. The revised estimated sensitivity for the AQP4‐IgG FCBA with these samples also included in the analysis would be 77.2% vs. the previously reported estimate of 71.6%. Performing a similar analysis to above (assuming again very conservatively that only ~90% would test positive by LCBA; i.e., 16 samples), the estimated sensitivity of AQP4‐IgG LCBA would be 95.7% vs. the previously reported estimate of 97.3%. Again, we note a modest increase in the sensitivity of AQP4‐IgG FCBA in this analysis, but there remains a marked discrepancy in estimated sensitivity between the two assays, and the same caveats discussed above regarding this being a “best‐case” scenario for the FCBA would apply.

## Author Contributions

All authors: drafting and revision of the manuscript for intellectual content.

## Conflicts of Interest

Y. Said, C. Tran, L. Rezavi, and P. Caturegli report no disclosures. E.P. Flanagan has served on advisory boards for Alexion, Genentech, Horizon Therapeutics, and UCB. He has received research support from UCB. He received royalties from UpToDate. Dr. E.P. Flanagan is a site principal investigator in a randomized clinical trial of Rozanolixizumab for relapsing myelin oligodendrocyte glycoprotein antibody‐associated disease run by UCB. Dr. E.P. Flanagan is a site principal investigator and a member of the steering committee for a clinical trial of satralizumab for relapsing myelin oligodendrocyte glycoprotein antibody‐associated disease run by Roche/Genentech. Dr. E.P. Flanagan has received funding from the NIH (R01NS113828). Dr. E.P. Flanagan is a member of the medical advisory board of the MOG project. Dr. E.P. Flanagan is an editorial board member of Neurology, Neuroimmunology, and Neuroinflammation, The Journal of the Neurological Sciences, and Neuroimmunology Reports. A patent has been submitted on DACH1‐IgG as a biomarker of paraneoplastic autoimmunity. Dr. E.S. Sotirchos has consulted for Alexion, Amgen, TG Therapeutics, and Roche/Genentech, has received speaker honoraria from Alexion, is site principal investigator for studies funded by Alexion, Roche/Genentech, UCB, and Ad Scientiam.

## Data Availability

The authors have nothing to report.
